# Clinical Competence of Neuroscience Nurses in Inpatient Wards and Intensive Care Units: A Mixed-Methods Systematic Review

**DOI:** 10.1097/JNN.0000000000000893

**Published:** 2026-05-05

**Authors:** Inkeri Hutri, Tiia Saastamoinen, Aki Laakso, Toni Haapa

**Affiliations:** 1 Inkeri Hutri, Faculty of Medicine, University of Helsinki, HUS Neurocenter, Helsinki University Hospital and Doctoral Programme in Clinical Research, Helsinki, Finland; 2 Tiia Saastamoinen, Metropolia University of Applied Sciences, Helsinki University Hospital and University of Helsinki, Helsinki, Finland; 3 Aki Laakso, University of Helsinki, HUS Neurocenter, HUS Helsinki University Hospital and Clinical Neurosciences, Helsinki, Finland; 4 Toni Haapa, University of Helsinki and Helsinki University Hospital, Helsinki, Finland; 5 Lovisenberg Diaconal University College, Oslo, Norway

## Abstract

**BACKGROUND:**

Neuroscience nurses need clinical competence (CC) when taking care of neuropatients. In this study the selected nursing context is inpatient wards and intensive care units (ICU) in university hospitals and tertiary referral centers where neuroscience patients are treated 24/7, patients’ conditions can change rapidly, and nurses execute clinical interventions continually for adult patients. The aim was to describe and synthesize the available information on the types of CC needed in neuroscience nursing (NSN) and to describe how the competencies are or should be ensured.

**METHODS:**

A mixed-method systematic review of literature from 2014 to 2024. Descriptive qualitative and quantitative studies were included in a convergent integrated approach. Qualitative synthesis was conducted through thematic inductive analysis of the combined data.

**RESULTS:**

Twenty studies met the inclusion criteria. CC in NSN in inpatient wards and ICUs was categorized into four main categories: neurospecific interventions, core nursing interventions, psychosocial interventions, and ensuring the quality and safety of patient care. Regarding how CC is ensured in clinical practice, the categories were postgraduate education, training sessions and feedback, extensive work experience and belonging to a neuroscience work community, and evidence-based materials and protocols. In terms of recommendations for ensuring CC, the categories identified were education and training in varying facilities, increasing knowledge and theoretical basis, and local opinion leaders, and the use of standardized tools.

**CONCLUSION:**

NSN requires specific and general CC in executing nursing interventions in hospital inpatients wards and ICUs. CC in NSN is ensured through various methods, and it will remain important to ensure and assess nurses’ competence to deliver high-quality and safe nursing care. This review emphasized the need to evaluate the CC of NSN in clinical practice.

Clinical competence (CC) in nursing can be defined as a combination of knowledge, skills, and attitudes that are consistent with those required for fieldwork. CC is gained over time through practice, repetition, and increasing experience. Nurses need to acquire personal, social, and professional competencies during their studies and work with support from their organization.^1^ CC is relevant in hospital environments because it affects patient safety and health outcomes as well as nurses’ job performance and satisfaction.^1–3^ NSNs provide care to various patients with special needs. Recovering does not necessarily mean a return to the functional level patients were at before diagnosis.^4^ It is estimated that more than three billion people worldwide are living with neurological conditions, which are now the leading cause of illness and disability worldwide.^5^

For this review the selected nursing context was inpatient wards and intensive care units (ICUs) in university hospitals and tertiary referral centers, where neuroscience patients are treated 24/7, patients’ conditions can change rapidly, and nurses execute clinical interventions continually for adult patients. No comprehensive review of CC in NSN has previously been conducted, and synthesized evidence is needed to support clinical nursing practice in the neuroscience context. The aim is to describe and synthesize the available information on the kinds of clinical competence (CC) that are needed in neuroscience nursing (NSN) and to describe how it is or should be ensured. The review aims to answer the research question: What kinds of clinical competence are needed in neuroscience nursing in inpatient wards or ICUs and what methods are used or should be used to ensure it?

## Methods

**FIGURE 1:**
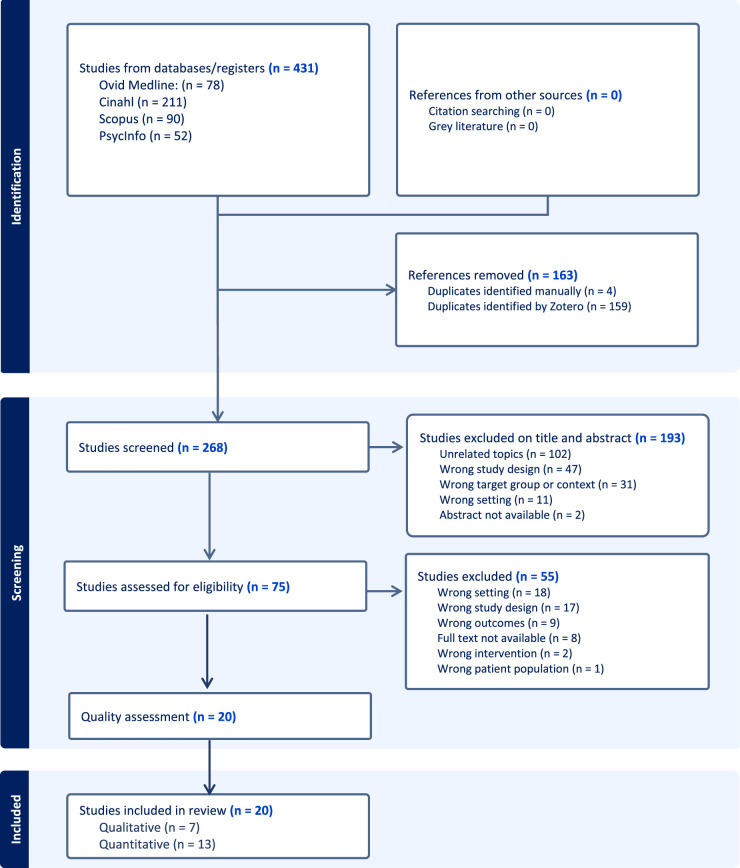
PRISMA Flowchart of the selection of included articles.

A mixed-methods systematic review was conducted according to the Joanna Briggs Institute guidelines for mixed-methods systematic reviews.^6^ The review is based on original studies using descriptive qualitative and quantitative methodologies. The review was registered in the PROSPERO database of the National Institute for Health Research. The PRISMA checklist was used while reporting this study.^7^ The literature was conducted from 2014 to 2024 using Ovid Medline, Scopus, CINAHL, and PsycInfo between February and April 2024. The keywords and search strategies are presented in Supplement Digital Content 1, http://links.lww.com/JNN/A659. MeSH-terms for the keywords were applied according to the requirements of each database. A library information specialist was consulted twice. Two researchers (IH and TS) independently conducted screening of titles, abstracts, and full texts with Covidence software. The inclusion and exclusion criteria were based on the population/concept/context format (see Table, Supplement Digital Content 1, http://links.lww.com/JNN/A659). The inclusion criteria were: registered nurses (Population); the clinical competence of neuroscience nurses (Concept); nursing in intensive care units or inpatient wards (Context); and qualitative, quantitative, or mixed study methods. The PRISMA flow diagram in Figure 1 shows the procedure used to search for and select studies.

The methodological quality of the 20 chosen studies was evaluated using the Critical Appraisal Checklist tool for qualitative research (7)^8^ and for analytical cross-sectional studies (13).^9^ Quality appraisal was performed independently by two reviewers (IH and TS) and any disagreements were resolved through discussion. No studies were excluded based on quality. The quality evaluation scores and extracted data are available in Supplemental Digital Content 2, http://links.lww.com/JNN/A660. One reviewer (IH) extracted data from the eligible studies. Quantitative data was transformed into a qualitative format (qualitizing).^10^ The two types of evidence were integrated using a convergent integrated approach combining quantitative data with qualitative data following transformation to gather descriptions about NSNs’ CC in inpatient wards and ICUs.^10,11^ ATLAS.ti 25 data analysis software was used to help code the literature data for analysis. From the meaning units, a total of 165 codes relating to the three research questions were generated. Qualitative synthesis involved thematic inductive analysis of the combined data. The data abstraction process proceeded by forming open codes for subcategories (69), which were further grouped into categories (22). The main categories (4) were only formed for the research question about CC in NSN.

## Results

The search found 431 titles. After applying inclusion and exclusion criteria, eliminating duplicates and irrelevant titles, and conducting quality appraisal, 20 articles were included in the analysis (see Supplemental Digital Content 2, http://links.lww.com/JNN/A660 for characteristics and quality scores). The original studies were conducted in 10 countries: China (2), Denmark (1), Korea (3), Netherlands (1), Poland (5), Singapore (1), Sweden (3), Turkey (1), UK (1), and USA (2). The sample sizes of qualitative studies ranged from 10 to 19 and those of quantitative studies ranged from 30 to 841 participants. The results of this review take into account only nurses working with neuropatients in inpatient wards and ICUs. CC in NSN was divided into 15 categories. These categories were organized into four main categories: clinical competence in neurospecific interventions, clinical competence in core nursing interventions, clinical competence in psychosocial support, and clinical competence in ensuring the quality and safety of patient care. The categories are presented in Table 1. Methods used to ensure CC in NSN were categorized into four categories, and recommended methods to ensure CC into two categories (see Supplemental Digital Content 3, http://links.lww.com/JNN/A661).

**TABLE 1:** Clinical Competence in Neuroscience Nursing

Clinical Competence in Neuroscience Nursing			
Clinical Competence in Neurospecific Interventions	Clinical Competence in Core Nursing Interventions	Clinical Competence in Psychosocial Support	Clinical Competence in Ensuring the Quality and Safety of Patient Care
• Assessing the level of consciousness • Monitoring and treating a state of confusion • Managing cerebrospinal fluid drainage care • Assessing patients’ swallowing and managing dysphagia	• Managing pharmacological and nonpharmacological care • Managing urinary tracts • Managing nutritional therapy • Monitoring and managing patients’ vitals (respiratory, hemodynamics, and pain) • Providing rehabilitative nursing and positioning patients • Ensuring asepsis and hygiene in patient care	• Providing informational and psychosocial support for the patients‘ family members • Providing informational and psychosocial support for patients • Performing palliative and end-of-life care	• Managing devices and tools in patient care • Managing nursing metrics, care planning, and following goals

The first category is *clinical competence in neurospecific interventions.* NSNs’ most prominent needs for specific CC were in assessing patients’ level of consciousness, neurological symptoms, nervous system, and possible changes in patients’ condition continually in clinical settings.^12–16^ On the basis of the reviewed studies, NSNs require CC in applying neurological and stroke assessment scales, of which the Glasgow Coma Scale (GCS) was the most mentioned.^12,13,15,17–19^ Documenting and reporting assessments is part of NSNs’ CC when assessing level of consciousness.^12^

Other neurospecific areas where NSNs need CC in nursing interventions include monitoring and treating a state of confusion in patients and deciding whether to use restraint, documenting this, and informing the patient about the restraining event. NSNs need CC regarding how to use physical and chemical restrain.^20–22^ CC is significant in preventing subsequent at-risk behaviors from becoming unmanageable and incidents from happening.^20,22^ CC in NSN also consists of knowledge about dysphagia, and competence in assessing patients’ swallowing and screening for possible dysphagia, which can occur after a stroke or other pathology affecting brain stem or lower cranial nerves.^16,23,24^ Managing the treatment and possible complications of dysphagia is an important neurospecific intervention, including assessing patients’ oral health, which requires CC.^16,24^ NSNs must have CC in applying and observing cerebrospinal fluid drainage with external ventricular drainage or with lumbar drainage. CC comprises skills in managing possible complications with drainage, when necessary.^14,25^

The second category is *clinical competence in core nursing interventions.* CC in NSN includes core nursing interventions such as those used in any nursing subspecialty, but with certain special features. On the basis of the reviewed studies, NSNs need CC in managing pharmacological and nonpharmacological care.^16,20,21,26,27^ Managing urinary tracts and managing nutritional therapies are also important.^16,24,26,28^ NSNs require CC when monitoring and managing patients’ vitals (including respiratory and hemodynamic), for pain-related nursing interventions,^14,16,22,23,28,29^ and to comprehensively ensure asepsis and hygiene in patient care.^28^ Providing rehabilitative nursing even in the ICU or inpatient ward, such as mobilizing and positioning patients, preventing adverse events, and monitoring patients’ physical abilities and functions, is also part of NSNs’ CC.^16,19,20^

The third category is *clinical competence in psychosocial support.* CC in NSN consists of providing psychosocial support both to patients and their family members. Psychosocial support can be seen as a clinical intervention because it involves providing informational guidance to patients and their family members, for which nurses require sufficient knowledge about the diagnosis and treatments.^16,21,26,30^ Other areas of CC needed in NSN are in communicating and collaborating with family members and providing them with emotional support.^16,30^ NSNs need CC in managing end-of-life care because nurses have a crucial role in observing the signs of impending death, which highlights the importance of palliative care knowledge and skills.^26^

The fourth category *is clinical competence in ensuring the quality and safety of patient care.* Managing devices, equipment, and tools used in clinical patient care is part of NSNs’ CC. Nurses use devices to monitor parameters and operate ECG^14^ and to manage different lines,^22^ drains,^22,25^ tubes, and implants.^22,26^ They also use catheters for cannulation^28^ and use restraining equipment.^20^ CC is needed in managing different nursing metrics, creating nursing plans for patients, and following treatment goals to facilitate patients’ recovery.^16,19,20,26^

Some reviewed articles described methods used to ensure NSNs’ CC. Mostly, NSNs participate in different kinds of training and specialization courses concerning neuroscience CC^15,19,25,27^ in the form of mandatory lectures, e-learning, or refresher courses with the possibility of receiving personal feedback.^12,20,28,31^ Some training is provided by organizations or departments^17,20,24^ and some at out-of-hospital congresses, conferences, or symposiums.^19,24,31^ Evidence-based protocols and guidelines which ensure NSNs’ CC exist nationally and organizationally.^15,20^ Some nurses undergo postgraduate education involving certification, such as with a diploma or MSc or BSc degree, which is also seen as ensuring CC.^17–19,25,28^ In two studies a connection was observed between ensuring competence and work experience.^23,28^ Working as a neurological nurse on a neurological ward was related to increased knowledge and CC, for example, in dysphagia and external ventricular drainage care, compared with working in other specialties.^23,25^ Professional titles, local opinion leaders, and sharing competence were also recognized as means to ensure CC.^15,20,25^

Nurses were also asked about recommended methods to ensure CC in NSN. Common responses included ongoing and regular education and training in different medical and neuro subjects, for example, principles of stroke care, vascular diseases, thrombectomy, issues in the field of psychotherapy, innovations in NSN, and coping with pain.^15,16,27–29,31^ Desired areas to increase knowledge included intensive neurosurgical therapy and administrating parenteral infusions.^14,31^ Learning facilities mentioned to ensure CC in future included web-based and simulation-based learning and learning by practice.^12,14,15^ Also, local opinion leaders should be a focus in terms of ensuring NSNs’ CC, and this can be facilitated by using standardized tools.^13,15^

## Discussion

Previous authors have described NSN CCs using similar terms to those in Table 1. Nursing skills, such as swallow screening, surgical drains, and mobilizations, are the essential components of NSNs’ orientation, which partly involve the same interventions as those examined in the reviewed studies.^32^ However, CC in NSN can also be examined from other perspectives, for example, in terms of different neuro diagnoses or disease groups.^32^ Neurological assessment as a neurospecific intervention is the foundational database NSNs use to detect neurological changes.^33^ GCS education should be a core requirement for nurses working with patients who require level-of-consciousness assessment.^34^ Frequent neurological and vital sign assessments are indicated to capture neurological deterioration and prevent secondary complications.^35^ Implementing evidence-based strategies in nursing interventions ensures nurses’ CC, for example, in preventing enteral nutritional complications, decreasing infection rates, administering treatments, monitoring metabolic markers, training staff, and involving families, and facilitating early patient mobility.^15,33,36,37^ Nurse stroke competencies are a hallmark of providing evidence-based care^35^ and nurses make substantial contributions, for example, to end-of-life care decision-making for patients with neurological diseases.^38^

On the basis of the studies included in this literature review, the impact of interventions designed to ensure CC, as well as their evaluation, is addressed only to a limited extent. The effectiveness of different kinds of educational interventions to ensure nurses’ CC in NSN has been reported in other studies outside the scope of this review, for example, in reduction of EVD-related ventriculitis.^39^ It should be noted that there are barriers to education and to professional development as well, such as lack of time and funding, lack of interest in topics, and preferences for certain training formats.^27,31^ There is also variance across Europe in postregistration neuroscience education and NSNs´ perceived educational needs.^40^ One of the chosen studies showed that extensive work experience correlates with better CC and one study found that the correct answer to a question regarding the use of protective gloves was more often chosen by the youngest nurses because of their more recent evidence-based knowledge.^23,28^

### Limitations

The search process was limited to publications from the years 2014–2024, and this has introduced selection bias, and the number of included articles was limited. Broader time frame would likely yield more results. Some of the included studies also targeted other health care professionals. No grey literature was used. None of the studies were excluded based on quality since all studies reached the cut-off value of 50%. Study screening and quality appraisal were conducted by two researchers, but data extraction and analysis were performed by one researcher, which may have increased the subjectivity of the results. The analysis was discussed by all authors. The results of this review examine the topic using descriptive methods, while it could be further explored using alternative methodological approaches. Randomized controlled trials and quasi-experimental studies were excluded from the review, which may have particularly affected the description of ensuring CC, as this topic has likely been more frequently studied using those research methods.

## Conclusion

Nurses working as NSN require neurospecific CC to provide effective and safe patient care. By describing the various nursing interventions associated with NSN, CC can be articulated in a more detailed and visible manner. There are other nursing interventions that could also relate to NSN CC which were not mentioned in this systematic review, for example assisting in different kinds of bedside procedures or managing other neurological symptoms and situations. Also, multidisciplinary cooperation is an important part of everyday NSN clinical practice, however, it did not emerge in this review. NSNs should be empowered to maintain and develop CC in inpatient wards and ICUs because NSN is continuously evolving, evidence-based knowledge is growing, and new models of clinical practice are implemented. Ensuring and assessing CC is essential for supporting multiple factors, including nursing leadership and organizational effectiveness for maintaining high-quality care, fostering professional development, and strengthening health care systems. On the basis of the results of this review, holistic assessment methods could be developed that integrate both neurospecific CC and core nursing competencies.

## Supplementary Material

## References

[1] Nabizadeh-Gharghozar Z Alavi NM Ajorpaz NM . Clinical competence in nursing: A hybrid concept analysis. Nurse Educ Today. 2021;97:104728. 33348301 10.1016/j.nedt.2020.104728

[2] Tura MR Mulu D Kadir A Getahun A Megersa Y . Nurse’s clinical competence and its associated factors among working in Ethiopia: a cross-sectional study. Sage Open Nurs. 2024;10:23779608241275213. 39697820 10.1177/23779608241275213 PMC11653466

[3] Zaitoun RA Said NB de Tantillo L . Clinical nurse competence and its effect on patient safety culture: a systematic review. BMC Nurs. 2023;22(1):173. 37208727 10.1186/s12912-023-01305-w PMC10196295

[4] Pearce SE Ethical perspectives and end-of-life care Hickey JV . The Clinical Practice of Neurological and Neurosurgical Nursing, 8th ed. Wolters Kluwer; 2019:48.

[5] GBD Nervous System Disorders Collaborators . Global, regional, and national burden of disorders affecting the nervous system, 1990-2021: a systematic analysis for the Global Burden of Disease Study 2021. Lancet Neurol. 2024;23(4):344–381. 38493795 10.1016/S1474-4422(24)00038-3 PMC10949203

[6] Lizarondo L Stern C Carrier J Chapter 8: Mixed methods systematic reviews. Aromataris E Lockwood C Porritt K Pilla B Jordan Z . JBI Manual for Evidence Synthesis. JBI; 2024.

[7] Page MJ McKenzie JE Bossuyt PM . The PRISMA 2020 statement: an updated guideline for reporting systematic reviews. Syst Rev. 2021;10(1):89. 33781348 10.1186/s13643-021-01626-4 PMC8008539

[8] Lockwood C Munn Z Porritt K . Qualitative research synthesis: methodological guidance for systematic reviewers utilizing meta-aggregation. Int J Evid Based Healthc. 2015;13(3):179–187. 26262565 10.1097/XEB.0000000000000062

[9] Moola S Munn Z Tufanaru C Chapter 7: Systematic reviews of etiology and risk. Aromataris E Munn Z . JBI Manual for Evidence Synthesis. JBI; 2020.

[10] Lizarondo L Stern C Salmond S . Methods for data extraction and data transformation in convergent integrated mixed methods systematic reviews. JBI Evid Synth. 2025;23(3):429–440. 39829248 10.11124/JBIES-24-00331

[11] Sandelowski M Voils CI Barroso J . Defining and designing mixed research synthesis studies. Res Sch. 2006;13(1):29. 20098638 PMC2809982

[12] Bae KS Roh YS . Training needs analysis of Korean nurses’ neurological assessment competency. Nurs Health Sci. 2020;22(1):99–107. 31609541 10.1111/nhs.12654

[13] Kerr RG Bacon AM Baker LL . Underestimation of pupil size by critical care and neurosurgical nurses. Am J Crit Care. 2016;25(3):213–219. 27134226 10.4037/ajcc2016554

[14] Lee JE Sim IO . Gap between college education and clinical practice: experience of newly graduated nurses. Nurs Open. 2020;7(1):449–456. 31871730 10.1002/nop2.409 PMC6917953

[15] Reynolds SS McLennon SM Ebright PR Murray LL Bakas T . Program evaluation of neuroscience competency programs to implement evidence-based practices. J Eval Clin Pract. 2017;23(1):149–155. 27766734 10.1111/jep.12654

[16] Tulek Z Poulsen I Gillis K Jonsson AC . Nursing care for stroke patients: a survey of current practice in 11 European countries. J Clin Nurs. 2018;27(3-4):684–693. 28815784 10.1111/jocn.14017

[17] Cook NF Braine ME Trout R . Nurses’ understanding and experience of applying painful stimuli when assessing components of the Glasgow Coma Scale. J Clin Nurs. 2019;28(21-22):3827–3839. 31343105 10.1111/jocn.15011

[18] Mattar I Liaw SY Chan MF . Nurses’ self-confidence and attitudes in using the Glasgow Coma Scale: a primary study. Nurs Crit Care. 2015;20(2):98–107. 24450732 10.1111/nicc.12077

[19] Ślusarz R . The use of clinimetrics in the practice of a neurosurgical nurse. preliminary reports. J Neurol Neurosurg Nurs. 2022;11(3):124–129.

[20] Tresfon J Langeveld K Brunsveld-Reinders AH Hamming J . Coming to grips—how nurses deal with restlessness, confusion, and physical restraints on a neurological/neurosurgical ward. Glob Qual Nurs Res. 2023;10:1–17. 10.1177/23333936221148816 PMC9880574 36712230

[21] Guenna Holmgren A Juth N Lindblad A von Vogelsang A . Nurses’ experiences of using restraint in neurosurgical care – a qualitative interview study. J Clin Nurs. 2022;31(15/16):2259–2270. 34514650 10.1111/jocn.16044

[22] Guenna Holmgren A von Vogelsang AC Lindblad A Juth N . Understanding nurses’ justification of restraint in a neurosurgical setting: a qualitative interview study. Nurs Ethics. 2023;30(1):71–85. 36266990 10.1177/09697330221111447 PMC9902980

[23] Skrzypek-Czerko M Zielińska M Roszmann A Jerzykowska M Nowakowska H . The level of knowledge about dysphagia among neurological and internal medicine nurses. J Neurol Neurosurg Nurs. 2021;10(1):10–17.

[24] Wang R Song Y He Y Long S Feng L . Status of knowledge, attitude and practice of poststroke dysphagia in neurological nurses in China: a cross-sectional study. PLoS One. 2023;18(4):e0284657. 37083919 10.1371/journal.pone.0284657 PMC10121028

[25] Sun Y Yu H Tian Y Zhang J Zhou J . A survey on the current status of adult external ventricular drainage care: exploring content framework and the need for group standards. Altern Ther Health Med. 2023;30(5):244–248. 37917907

[26] Eriksson H Andersson G Olsson L Milberg A Friedrichsen M . Ethical dilemmas around the dying patient with stroke: a qualitative interview study with team members on stroke units in Sweden. J Neurosci Nurs. 2014;46(3):162–170. 24796473 10.1097/JNN.0000000000000049

[27] Ślusarz R Filipska K . Postgraduate education of neurological nurses -- preliminary reports. J Neurol Neurosurg Nurs. 2020;(2):71–75.

[28] Sternal D Franek G Pieńkus D . Knowledge of Nurses on Prevention of Nosocomial Infections in post-stroke Patients. J Neurol Neurosurg Nurs. 2014;3(2):58–63.

[29] Kang H Roh YS . Needs assessment survey for stroke care core competency-based training for neuroscience nurses. J Contin Educ Nurs. 2024;55(2):63–68. 37921480 10.3928/00220124-20231030-02

[30] Guldager R Loft MI Poulsen I . Neuroscience nurses’ comprehension of collaborating with and involvement of relatives: a qualitative interview study. Nordic J Nurs Res. 2022;42(3):133–139.

[31] Ślusarz R . Professional development of neurosurgical nurses. Preliminary reports. J Neurol Neurosurg Nurs. 2019;8(3):119–123.

[32] Vyas MB Bautista C Daniels L Guanci MM Rhudy L . The essential components of adult critical care neuroscience nursing orientation: a Delphi study. J Neurosci Nurs. 2025;57(1):3–8. 39602519 10.1097/JNN.0000000000000807

[33] Figueiredo R Castro C Fernandes JB . Nursing interventions to prevent secondary injury in critically ill patients with traumatic brain injury: a scoping review. J Clin Med. 2024;13(8):2396. 38673667 10.3390/jcm13082396 PMC11051360

[34] Cook N Trout R Waterhouse C . The Glasgow Coma Scale: an international standard for education and practice with adults. Br J Neurosci Nurs. 2025;21(Sup1c):S1–S36.

[35] Greenberg SM Ziai WC Cordonnier C . 2022 Guideline for the Management of Patients With Spontaneous Intracerebral Hemorrhage: A Guideline From the American Heart Association/American Stroke Association. Stroke. 2022;53(7):e282–e361. 35579034 10.1161/STR.0000000000000407

[36] Broadway K Nuila CM . Implementation of an interprofessional mobility program in a neurosurgical intensive care unit. J Neurosci Nurs. 2023;55(6):205–210. 37738106 10.1097/JNN.0000000000000729

[37] Jiao J Chen Y Yang L . Nursing practice based on evidence-based concepts to prevent enteral nutrition complications for critically ill neurosurgical patients. Front surg. 2022;9(101645127):857877. 35372491 10.3389/fsurg.2022.857877 PMC8971188

[38] Jonsdottir G Vilhjalmsson R Sigurdardottir V Hjaltason H Klinke ME Jonsdottir H . Nursing contribution to end-of-life care decision-making in patients with neurological diseases on an acute hospital ward: documentation of signs and symptoms. BMC Nurs. 2025;24(1):271. 40069809 10.1186/s12912-025-02897-1 PMC11899557

[39] Reiter LA Taylor OL Jatta M . Reducing external ventricular drain associated ventriculitis: an improvement project in a level 1 trauma center. Am J Infect Control. 2023;51(6):644–651. 36116678 10.1016/j.ajic.2022.08.029

[40] Braine ME Cook N . An evaluation of post-registration neuroscience focused education and neuroscience nurses’ perceived educational needs. Nurse Educ Today. 2015;35(11):1069–1074. 26116030 10.1016/j.nedt.2015.05.021

