# Supplementary material for: Clinical Competence of Neuroscience Nurses in Inpatient Wards and Intensive Care Units: A Mixed-Methods Systematic Review
Source: J Neurosci Nurs. 2026 May 5;58(4):175–80. doi: 10.1097/JNN.0000000000000893 (PMC13317913; doi:10.1097/JNN.0000000000000893)
Supplement: Supplementary file 1 [file jnn-58-175-s001.docx]

Supplement Digital Content 1. Search strategies for the systematic mixed methods review with inclusion and exclusion criteria.

| **PCC** | **Inclusion criteria** | **Exclusion criteria** | **Keywords** |
| --- | --- | --- | --- |
| Population | Neuroscience nurses | Other health care professionals, nursing students, nurse managers | neuroscience nurs* OR neuronurs* OR neurological nurs* OR neurosurgical nurs* OR neuro nurs* |
| Concept | Clinical competence, ensuring clinical competence | Other competence areas | competenc* OR professional competenc* OR skill* OR know* OR expertise* OR proficiency* OR qualificatio* OR capability* OR capacity* OR ability* |
| Context | Intensive care unit, inpatient ward | Pediatric/neonatal patients, rehabilitation, ER, OR (inc. pre-, intra-, and post), home care, psychiatric care, senior care, outpatient care, spinal cord injuries |  |
| Study design | Qualitative and quantitative and mixed methods | Literature reviews, systematic reviews, case studies, editorial texts, quality improvement projects, quasi-experimental studies, randomized controlled trials, prevalence studies |  |
| Search limitations | Peer reviewed, published 2014 -2024, full text available, language English, Finnish or Swedish |  |  |

Note. PCC = population, concept, context
