# Supplementary material for: Clinical Competence of Neuroscience Nurses in Inpatient Wards and Intensive Care Units: A Mixed-Methods Systematic Review
Source: J Neurosci Nurs. 2026 May 5;58(4):175–80. doi: 10.1097/JNN.0000000000000893 (PMC13317913; doi:10.1097/JNN.0000000000000893)
Supplement: Supplementary file 2 [file jnn-58-175-s002.docx]

Supplement Digital Content 2. Table of eligible studies (n=20).

| **Author(s),**  **year, country** | **Aim of the study** | **Participants** | **Methodology, data collection, and**  **data analysis** | **Key findings** | **Quality consensus points** |
| --- | --- | --- | --- | --- | --- |
| Eriksson et al. (2014), Sweden | To study ethical dilemmas regarding dying stroke patients in acute stroke units, approaches to these dilemmas, and their consequences for healthcare professionals. | 13 nurses in three hospitals (1 university hospital and 2 general hospitals). | Qualitative study with three focus group interviews.  Content analysis. | Nurses described ethical dilemmas in stroke care, particularly around life-support decisions, nutrition strategies, and palliative care. They felt that patients in palliative phases were sometimes overtreated, leading to confusion and emotional distress for both staff and families. Conflicts among family members and unclear communication responsibilities further complicated care, leaving nurses uncertain about whom to inform and how to honor patient wishes. | 8/10^1^ |
| Sternal et al. (2014), Poland | To assess nurses’ knowledge about nosocomial infections and the procedures used most often with post-stroke patients. | A group of 80 nurses working in randomly selected stroke and neurological units in Silesia province. | Diagnostic survey with a specially developed questionnaire based on literature. Descriptive statistical analysis to test the frequency of answers within age groups according to seniority, education, and dependence between these with a chi-square test was used. | Nurses had received training on nosocomial infections and recognized its importance, but significant knowledge gaps remained. Many respondents gave incorrect definitions of infection and lacked understanding of procedures related to catheter and airway management. Nurses with more education demonstrated better knowledge and hygiene practices, though 25% admitted not consistently following handwashing protocols. Equipment shortages and inconsistent use of protective gear were also noted, with younger nurses more accurately identifying proper glove-use procedures compared to older age groups. | 5/8^2^ |
| Mattar et al. (2015), Singapore | To examine nurses’ self-confidence and attitudes towards the Glasgow Coma Scale (GCS). | 114 registered nurses in one acute care hospital. | Descriptive correlational study with questionnaire. The descriptive statistics mean and standard deviation were used to analyze background variables. Analysis of variance (ANOVA) and independent t-tests were used to examine differences between each factor within each scale. Factors with p < 0.05 in the univariate analysis were included in the multiple regression (stepwise) analyses. Variance inflation factors were used to examine the collinearity between independent variables on the regression model, and the Shapiro-Wilk test was used to examine the normality of the residual of the regression model. | Nurses’ attitudes toward using the GCS were significantly influenced by their clinical discipline, years in nursing, and time spent in neuroscience settings. A strong positive correlation was observed between nurses’ attitudes and their self-confidence in using the GCS. Nurses with more experience in neuroscience care were more confident and better able to apply the GCS to complex patient situations. This suggests that both experience and positive attitudes contribute to greater competence in neurological assessments. | 8/8^2^ |
| Kerr et al. (2016), USA | To evaluate nurses’ abilities to assess pupil diameter accurately and detect unequal pupils~~:~~ and to determine the accuracy of current practice, specify the thresholds at which the quality of subjective pupil measurement degrades, and examine nurses’ ability to detect sluggish and unequal pupils. | Phase 1: 30 critical care and neurosurgical nurses.  Phase 2: 27 critical care and neurosurgical nurses.  Phase 3: 489 assessments of 93 patients. | Threepart observational study included assessment of drawings of eyes with an iris and pupil, examination of photographs of human eyes, and bedside examination of patients with a head injury.  Descriptive statistics were analyzed such as means (standard deviations) for continuous variables and counts (percentages) for categorical variables. Correlations were assessed with Pearson correlation coefficients. | Nurses’ accuracy in measuring pupil diameter decreased as the pupil size increased, with underestimations reaching up to 1.4 mm for larger pupils. Consistency in measurements was low, with only about half of duplicated assessments matching, and just 11.7% of repeated photo evaluations being both consistent and correct. Nurses’ bedside assessments showed notable rates of false positives and negatives in assessing pupil reactivity. | 6/8^2^ |
| Reynolds et al. (2016), USA | To evaluate the usefulness of two competency programs that sought to improve neurocritical nurses’ knowledge of and adherence to evidence-based stroke and spinal cord injury guidelines. | 10 neurocritical nurses who participated in both Stroke and Spinal Cord Injury Competency Programs. | Qualitative research evaluating implementation outcomes with semi-structured, one-on-one interviews by telephone.  The transcripts were deductively analyzed and categorized using a predetermined implementation outcomes framework. | Nurses found the strategies—local opinion leaders, printed educational materials, and educational outreach—valuable, especially the outreach sessions and printed materials. Suggestions for improvement included hands-on practice with assessments. The programs were seen to enhance guideline adherence and nursing knowledge, with recommendations for ongoing education, reminders, and ongoing support from local opinion leaders to sustain improvements. | 9/10^1^ |
| Tulek et al. (2018), Turkey | To conduct a survey of clinical nursing practice in European countries in accordance with the European Stroke Strategies 2006 and to examine to what extent the European Stroke Strategies have been implemented in stroke care nursing in Europe. | 92 nurses working in stroke care from 11 European countries. | Descriptive cross-sectional survey with a questionnaire.  Analysis using descriptive statistics including mean, standard deviation, range, number, and percentage. | Most nurses reported monitoring patients’ consciousness and physical abilities within the first 48 hours after stroke onset and mobilizing them once stable. Bedside swallowing assessments were commonly performed within 24 hours. Urinary care practices varied, with 42% using permanent catheters and 85% measuring residual urine volume. Nurses also frequently assessed eating ability, oral health, and pain, and provided psychosocial support and written information to patients and families during acute care. | 6/8^2^ |
| Cook et al. (2019), UK | To evaluate nurses’ application, understanding, and experience of applying painful stimuli when assessing components of the Glasgow Coma Scale. | 273 nurses practicing within a neuroscience nursing setting (members of BANN/EANN or working in UK neuroscience units). | Descriptive online self‐reported survey design.  Descriptive statistics such as mean and standard deviation were used to analyze background variables. | Most nurses had received neuroscience education but 31.1% had no formal education in the field. Painful stimuli were typically applied for less than 15 seconds, with trapezius pinch being the most common technique for assessing eye-opening and motor responses. Technique choices were often based on training or guidelines, but complications such as bruising and even serious injuries were reported, especially with sternal rub and supraorbital pressure. While most nurses assessed eye and motor responses simultaneously to reduce discomfort, over half (54.6%) expressed concerns about the use of painful stimuli, highlighting the need for clearer protocols and safer practices. | 7/8^2^ |
| Lee & Sim (2019), Korea | To examine the experience of new nurses working on a neurology ward and the differences between their training and hospital practice. | 12 newly graduated nurses working on a neurology ward (with less than year of clinical experience in a university hospital). | Qualitative focus group interviews; three groups with 3-4 people in each.  Content analysis method. | New nurses in neurology wards reported significant challenges due to ineffective theoretical education and difficulty applying nervous system knowledge to clinical practice. They struggled with basic assessments and procedures, such as the Glasgow Coma Scale and drainage management, highlighting a gap between theory and real-world application. | 7/10^1^ |
| Slusarz (2019), Poland | To analyze the professional development of neurosurgical nurses. | 93 nurses employed in 6 neurosurgical centers. | Diagnostic survey with a questionnaire.  The hypotheses were verified based on the U Mann–Whitney test and the Kruskal–Wallis test. The issues of pre- and post-graduate education and professional experience were analyzed in comparison with selected sociodemographic variables. | The most popular forms of postgraduate education among nurses were refresher courses and participation in congresses or conferences. Most training was funded privately or co-financed by workplaces, with professional organizations rarely contributing. Common barriers to professional development were lack of time, uninteresting topics, and financial constraints. | 4/8^2^ |
| Bae & Roh (2020), Korea | To identify nurses' competency levels in performing neurological assessments and prioritize their related training needs using importance–performance analysis. | 213 nurses working in an ICU (n=95), emergency department (n=68), or neurological ward (n=50). | Descriptive, cross-sectional survey study with a specially developed self-administered web-based questionnaire.  Responses were analyzed using descriptive statistics. The construct validity of the scale was investigated using exploratory factor analysis, the mean difference  between importance and performance was analyzed using the paired t-test, and comparison of competency among ward, ICU and emergency department nurses was analyzed using one way analysis of variance (ANOVA). | The analysis revealed seven key components of neurological assessment competency, accounting for 70.34% of the variance. Across all factors, nurses rated performance significantly lower than importance, with the largest gap in "signs and symptoms" and the smallest in "assessment of an unconscious patient." Although 57.9% had prior training—mostly through lectures—simulation-based learning was the most preferred method. | 7/8^2^ |
| Slusarz & Filipska (2020), Poland | To analyze the postgraduate education of neurological nurses. | 128 nurses employed in 9 neurological centers. | Diagnostic survey using a questionnaire.  Analysis using elements of descriptive statistics. The Mann–Whitney and Kruskal–Wallis tests were used to statistically compare the means of individual groups. | Specialization was the preferred form of training among nurses (61.7%), followed by specialist courses and conferences. Most postgraduate education was self-funded (93.8%), and while some nurses reported training specific to neurological nursing, many were unsure if such training existed. Key training needs identified included stroke care techniques, innovations in neuro-nursing, and communication and psychotherapy skills. Barriers to education included lack of time and funding, and preferences for training formats. | 4/8^2^ |
| Skrzypek-Czerko et al. (2021), Poland | To measure~~/~~ and assess the level of knowledge about dysphagia among nurses on neurological and internal medicine wards. | 70 nurses working on neurological and internal medicine wards. | Descriptive diagnostic survey with a questionnaire.  Analysis with statistical calculations. The relationship between “experience” and “test score” was measured with the Pearson correlation test. The Wilcoxon test was used to determine the relationship between “test score” and “workplace”. The Kruskal–Wallis test was performed to determine the relationship between “test score” and “education”. | Nurses working in neurological departments had significantly higher knowledge about dysphagia, especially regarding its definition, causes, and complications. Seniority also positively influenced knowledge levels, with experienced nurses more accurately identifying stroke as the most common cause and aspiration pneumonia as a serious complication. Neurological nurses demonstrated strong understanding of dietary recommendations and aspiration prevention, though some areas still require targeted education. | 6/8^2^ |
| Guenna Holmgren et al. (2022), Sweden | To describe nurses’ experiences of using restraint in neurosurgical care. | 15 nurses working in three neurosurgical departments (with experience using restraint). | Qualitative, descriptive design guided by naturalistic inquiry. Data collection via interviews.  Inductive qualitative content analysis (COREQ reporting guidelines). | The overarching theme was "the struggling professional", highlighting the emotional and practical challenges faced by nurses when using restraint in clinical care. Nurses experienced internal conflict, feeling that restraint contradicted their values regarding patient dignity and autonomy, especially when used on frightened or elderly patients. Restraint decisions were often made individually without clear guidelines, leading to inconsistency and distress. Nurses also reported a lack of support from physicians and colleagues, unclear responsibilities, and inadequate documentation and communication practices surrounding restraint use. | 9/10^1^ |
| Guldager et al. (2022), Denmark | To illuminate neuroscience RNs’ understanding of collaborating with and involving relatives. | 19 registered neuroscience nurses from 3 wards in one university hospital. | Qualitative study with hermeneutic design. Individual semi-structured interviews. Inductive thematic analysis inspired by Braun and Clarke. | Nurses emphasized the importance of collaboration with patients’ relatives but often confused providing information with genuine involvement. While they recognized the need for empathy and open dialogue, time constraints and lack of clear guidelines made meaningful engagement difficult. Relatives were seen as both valuable resources and potential burdens. Personal experience helped nurses better understand and support relatives, but structural challenges like understaffing and unclear leadership hindered care. | 7/10^1^ |
| Slusarz (2022), Poland | To analyze neurosurgical nurses’ use of measurement tools in practice. | 93 nurses employed in 6 neurosurgical centers. | Diagnostic survey technique with questionnaire.  In the descriptive statistical analysis of the material, the χ2 test of independence was used to verify the hypotheses regarding the existence of a relationship between the variables under study. | Most nurses (73%) reported using scales to assess patient condition, with the Glasgow Coma Scale (GCS) being the most used tool for neurosurgical patients. Other frequently used scales included those for pressure sore prevention and functional status assessment. Nurses with higher education and specialization were significantly more likely to use tools in clinical practice. | 4/8^2^ |
| Guenna Holmgren et al. (2023), Sweden | To understand nurses’ justifications for restraint use in neurosurgical care. | 15 nurses working in three neurosurgical departments (with experience of using or deciding on restraint in a neurosurgical department). | Qualitative descriptive design guided by naturalistic inquiry. Semi-structured interviews.  Inductive conventional content analysis. | There were three main categories explaining nurses’ use of restraint: patient-related factors, specific justifications, and general reasoning. Restraint was often applied to patients with acute neurosurgical conditions who were confused, restless, and required invasive devices. Nurses justified restraint primarily to protect patients from self-harm but also acknowledged using it for staff safety and convenience, especially when time and resources were limited. Decisions were typically made using a consequentialist approach, weighing the benefits against potential suffering, and preferences varied between physical and chemical restraint depending on perceived risks and ethical concerns. | 9/10^1^ |
| Sun et al. (2023), China | To investigate the level of adult external ventricular drainage care among neurosurgical nurses, delineate nursing priorities and challenges, and explore the necessity for establishing standardized guidelines for external ventricular drainage care. | A total of 841 valid responses from certified registered neurosurgical nurses working in 20 neurosurgery hospitals (minimum 1 year experience working in neurosurgery). | Multicenter cross-sectional study with an electronic questionnaire was used.  Descriptive statistical analysis such as means and standard deviations were employed for normally distributed measurement data. To compare different influencing factors, t tests or analysis of variance (ANOVA) were applied. | There were significant knowledge gaps among nursing staff regarding external ventricular drain (EVD) management, particularly in understanding cerebrospinal fluid volume, drainage rate, and complication handling. Factors such as education level, professional title, department, and prior training influenced nurses’ knowledge, with those in neurosurgical units showing stronger understanding. Ventriculostomy-related infections were identified as the most common complication, emphasizing the need for precise EVD care. | 6/8^2^ |
| Tresfon et al. (2023), Netherlands | To perform an extensive collaborative study to understand how restraint use guidelines and protocol relate to and support actual nursing practice on a neurological/neurosurgical ward, and to what extent nursing practice already incorporates safe aspects of dealing with physical restraints. | 15 nurses on a combined neurological and neurosurgical ward in a tertiary hospital were interviewed. Observational data was collected through 10 participants. | Qualitative ethnographic FRAM-method. Participant observations, single and group interviews, and an extensive member check were conducted. The observations and interviews formed the main inputs for the development of the FRAM model. | Nurses often relied on collaborative, experience-based practices to manage restlessness and confusion in patients, which helped prevent the need for physical restraints. When restraint was necessary, decisions were made collectively with attention to preserving patient freedom and consulting physicians and families. Despite mandatory training, protocol was rarely used in practice. | 9/10^1^ |
| Wang et al. (2023), China | To explore knowledge, attitudes, and practice (KAP) related to post-stroke dysphagia among neurological nurses in China. | 707 nurses with a nurse qualification certificate, working in a Department of Neurology and managing stroke patients with dysphagia. | Cross-sectional study design with a questionnaire.  Data with normal distribution and homogeneity of variance were expressed as the mean ± standard deviation (x ± s). Comparisons between two groups were performed through a t test, and comparisons between multiple groups were performed through analysis of variance. Descriptive data were expressed as frequencies, composition ratios (%), or percentages (%). Spearman correlation analysis was used between continuous variables and nursing knowledge, belief, and practice scores on dysphagia. Univariate analysis was used for other classified independent variables. | Nurses demonstrated moderate knowledge and practice levels regarding dysphagia after stroke, with notable gaps in areas such as screening, nutrition assessment, and complication management. Although attitudes toward dysphagia care were generally positive, training was limited. Nurses expressed strong interest in further training, especially in swallowing dysfunction. Factors such as age, education level, professional title, and neurology experience significantly influenced knowledge and practice. | 8/8^2^ |
| Kang & Young (2024), Korea | To determine priorities for competency-based training using importance–performance analysis of stroke care core competency among neuroscience nurses. | 154 neuroscience nurses who were directly in charge of caring for stroke patients in hospitals. | Cross-sectional descriptive survey with a web-based self-administered questionnaire.  Descriptive statistics were calculated to summarize quantitative data and paired t tests were used to calculate mean differences between perceived importance and performance scores. | Nurses rated the importance of stroke nursing practices significantly higher than their actual performance, with mean scores of 3.26 and 2.72 respectively. The greatest gap was in the principles of stroke care, while skin care showed the smallest difference. These results suggest that although nurses are aware of best practices, their self-rated performance remains low. Key areas identified for targeted training included stroke care principles and managing cardiovascular and respiratory effects. | 7/8^2^ |

¹Qualitative study = Quality score on JBI (Joanna Briggs Institute) critical appraisal checklist, which includes 10 criteria to assess the methodological quality of qualitative studies.

²Cross-sectional study = Quality score on JBI (Joanna Briggs Institute) critical appraisal checklist, which includes 8 criteria to assess the methodological quality of cross-sectional studies.
