# Supplementary material for: Clinical Competence of Neuroscience Nurses in Inpatient Wards and Intensive Care Units: A Mixed-Methods Systematic Review
Source: J Neurosci Nurs. 2026 May 5;58(4):175–80. doi: 10.1097/JNN.0000000000000893 (PMC13317913; doi:10.1097/JNN.0000000000000893)
Supplement: Supplementary file 3 [file jnn-58-175-s003.docx]

Supplemental Digital Content 3. Ensuring clinical competence in neuroscience nursing.

| METHODS USED TO ENSURE CLINICAL COMPETENCE IN NEUROSCIENCE NURSING |
| --- |
| - Postgraduate education - Training sessions and feedback - Extensive work experience and belonging to a neuroscience work community - Evidence-based materials and protocols |
| **RECOMMENDED METHODS TO ENSURE CLINICAL COMPETENCE IN NEUROSCIENCE NURSING** |
| - Education and training in varying facilities increasing knowledge and theoretical basis - Local opinion leaders and the use of standardized tools |
